# Supplementary material for: Transcriptomic Insights into Mechanisms of Early Seed Maturation in the Garden Pea (Pisum sativum L.)
Source: Cells. 2020 Mar 23;9(3):779. doi: 10.3390/cells9030779 (PMC7140803; doi:10.3390/cells9030779)

Zhewan−1,25(4)

Global Summary

%DE = 0.27

genes with fdr < 0.2 = 7213 (3426 + / 3787 −)

genes with fdr < 0.1 = 6566 (3118 + / 3448 −)

genes with fdr < 0.05 = 6145 (2918 + / 3227 −)

genes with fdr < 0.01 = 5423 (2602 + / 2821 −)

genes in genesets = 0

<FC> = 0

<t−score> = 0

<p−value> = 0

<fdr> = 0.73

Global Genelist

Rank	ID	log(FC)	p−value / fdr / Metagene	Description
1	TRINITY_DN3	0.03	0.0000000[illegible]00002	
2	TRINITY_DN2	−0.06	0.0000000[illegible]00002	
3	TRINITY_DN1	−0.05	0.0000000[illegible]00002	
4	TRINITY_DN4	−0.15	0.0000000[illegible]00002	
5	TRINITY_DN3	0.08	0.0000000[illegible]00002	
6	TRINITY_DN3	−0.05	0.0000000[illegible]00002	
7	TRINITY_DN3	−0.07	0.0000000[illegible]00002	
8	TRINITY_DN1	−0.19	0.0000000[illegible]00002	
9	TRINITY_DN1	−0.24	0.0000000[illegible]00002	
10	TRINITY_DN1	−0.06	0.0000000[illegible]00002	
11	TRINITY_DN1	0.25	0.0000000[illegible]00002	
12	TRINITY_DN1	−0.09	0.0000000[illegible]00002	
13	TRINITY_DN1	0.04	0.0000000[illegible]00002	
14	TRINITY_DN1	0.89	0.0000000[illegible]00002	
15	TRINITY_DN1	−0.04	0.0000000[illegible]00002	
16	TRINITY_DN5	−0.04	0.0000000[illegible]00002	
17	TRINITY_DN5	−0.05	0.0000000[illegible]00002	
18	TRINITY_DN5	0.15	0.0000000[illegible]00002	
19	TRINITY_DN1	0.32	0.0000000[illegible]00002	
20	TRINITY_DN1	0.53	0.0000000[illegible]00002	

Portrait

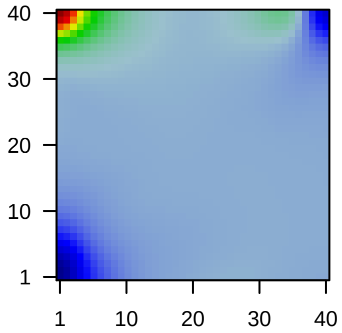

Top 100 DE genes

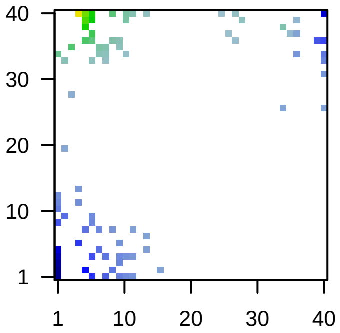

p−values

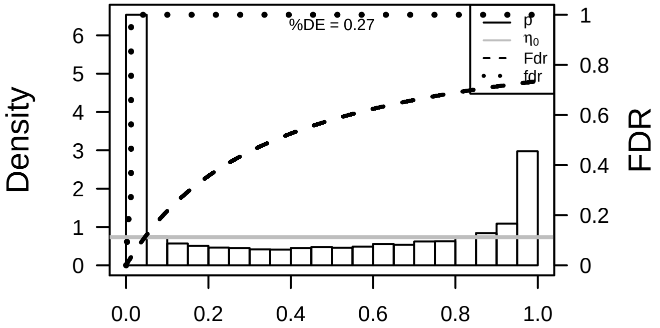

Supplement: Supplementary file 1 [file cells-09-00779-s001.zip › Supplementary materials/FileS2.zip!/FileS1/Summary Sheets - Samples/Zhewan.1.25.4..pdf]
